# Congenital Tufting Enteropathy: Biology, Pathogenesis and Mechanisms

**DOI:** 10.3390/jcm10010019

**Published:** 2020-12-23

**Authors:** Barun Das, Mamata Sivagnanam

**Affiliations:** 1 Department of Pediatrics, University of California, San Diego, La Jolla, CA 92093, USA; bdas@health.ucsd.edu; 2 Rady Children’s Hospital, San Diego, CA 92123, USA

**Keywords:** congenital tufting enteropathy, pathogenesis, EpCAM, intestinal epithelial dysplasia

## Abstract

Congenital tufting enteropathy (CTE) is an autosomal recessive disease of infancy that causes severe intestinal failure with electrolyte imbalances and impaired growth. CTE is typically diagnosed by its characteristic histological features, including villous atrophy, crypt hyperplasia and focal epithelial tufts consisting of densely packed enterocytes. Mutations in the EPCAM and SPINT2 genes have been identified as the etiology for this disease. The significant morbidity and mortality and lack of direct treatments for CTE patients demand a better understanding of disease pathophysiology. Here, the latest knowledge of CTE biology is systematically reviewed, including clinical aspects, disease genetics, and research model systems. Particular focus is paid to the pathogenesis of CTE and predicted mechanisms of the disease as these would provide insight for future therapeutic options. The contribution of intestinal homeostasis, including the role of intestinal cell differentiation, defective enterocytes, disrupted barrier and cell–cell junction, and cell-matrix adhesion, is vividly described here (see Graphical Abstract). Moreover, based on the known dynamics of EpCAM signaling, potential mechanistic pathways are highlighted that may contribute to the pathogenesis of CTE due to either loss of EpCAM function or EpCAM mutation. Although not fully elucidated, these pathways provide an improved understanding of this devastating disease.

## 1. Introduction

According to the World Health Organization (WHO), diarrhea remains a leading cause of malnutrition and the second leading cause of mortality for children under the age of five. Unlike the more treatable causes of diarrhea, congenital diarrhea and enteropathies (CODEs) are hereditary, monogenic disorders causing persistent, severe, chronic diarrhea in infants and often lead to life-threatening intestinal failure [1]. Patients with CODES, particularly those with epithelial cell defects, present in the first few months of life [2]. Epithelial cell defects are a hallmark of CODEs, and they share common pathophysiology, including defects in epithelial transporters, enzymes and metabolism, epithelial trafficking and polarity, enteroendocrine cell dysfunction, and/or immune cell dysregulation. Along with microvillus inclusion disease, a severe disorder belonging to the defective enterocyte trafficking and polarity category of CODEs is congenital tufting enteropathy (CTE). CTE, also known as intestinal epithelial dysplasia, is a rare, autosomal recessive disease of infancy presenting with profuse watery diarrhea, electrolyte imbalances, and impaired growth [3,4]. CTE is one of several intractable diarrheal diseases of infancy, with an incidence estimated at 1/50,000–100,000 live births in western Europe [3], although the incidence is higher in the Middle East [5]. Due to profuse diarrhea, with both an osmotic and secretory component, patients suffer from intestinal failure necessitating parenteral nutrition and, in some cases, intestinal transplant, both with inevitable complications such as liver disease, infection, rejection, and vascular complications [6]. Moreover, most patients never achieve enteral autonomy [7]. The diagnosis of CTE is based on characteristic structural changes in the small intestinal epithelium. Typical findings include partial or total villous atrophy and crypt hyperplasia, but the most characteristic pathologic abnormalities are focal epithelial tufts [8]. CTE or epithelial dysplasia was reportedly identified in 1994 [4], though a historically similar disease phenotype was first reported in 1978 [9], where it was termed as familial enteropathy. The patients with familial enteropathy reported having persistent diarrhea with villous atrophy, crypt hyperplasia, without an increase of inflammatory cells in laminar propria, absence of brush-border villous enterocytes and were dependent on total parenteral nutrition [9]. As the term “familial enteropathy” suggested, the patients had a family connection, with the disease reported in two patients who were products of a consanguineous marriage [9], confirming the hereditary nature of CTE. Mutations in epithelial cell adhesion molecule (EPCAM) were identified as the primary cause for CTE [10]. Although Serine Peptidase Inhibitor Kunitz Type 2 (SPINT2) mutations have also been reported in a syndromic form of the disease, most CTE patients possess mutations in EPCAM [11]. Though not all patients are reported in the literature, a recent review suggests 74% of those reported have mutations in EPCAM, while 26% have mutations in SPINT2 [12].

The significant morbidity and mortality and lack of direct treatments for patients with CTE results in an unmet need for effective therapeutic options, which require a better understanding of disease pathophysiology. The rare incidence of CTE and limitation of related studies have caused the disease to remain elusive, leading to poor prognosis and low quality of life. The current review will discuss the latest knowledge in CTE biology, including its clinical aspects, genetics, disease model systems and future therapeutics options. A particular focus will be on the pathophysiology. Moreover, this review also aims to highlight predicted or probable mechanisms of this disease based on the known dynamics of EpCAM and CTE models.

### 1.1. Clinical Aspects of CTE

CTE is generally diagnosed by esophagogastroduodenoscopy, noting duodenal histological abnormalities. The tufts are composed of disorganized enterocytes with focal crowding at villous tips and basement membrane abnormalities resulting in teardrop-like configurations. The severity of villous atrophy varies among patients. Although typically, no inflammatory cell infiltration is seen in CTE, some inflammatory infiltration can be found [13]. Just over 200 cases have been reported for this rare disease, and many of these are clinically heterogeneous [12]. The severity of this disease varies. Some patients need total parenteral nutrition, while others may need little parenteral support [14]. Analysis of the family history of CTE patients reveals that, as expected, siblings or cousins of CTE patients may be affected though parents/ancestors of the patients are often unaffected with no similar family history [10,14,15,16]. Though the majority of the cases have secretory diarrhea [17], some evidence suggests osmotic diarrhea in CTE as well [18]. Watery diarrhea is abundant in CTE patients irrespective of whether the infant is fed or fasted [19], although in some cases, fasting improves stool output [20]. Nutrient malabsorption and intractable diarrhea lead infants to become irritable and develop dehydration as well as weight loss [21], ultimately resulting in impaired growth and failure to thrive [3,10]. Apart from intractable diarrhea, some CTE patients develop vomiting and abdominal distension [12,21]. Elevated osmolality, osmotic gap and quantitative fecal fat have been reported in CTE [20,21], and equivalent fecal loss of Na+ was found when compared to congenital sodium diarrhea [22]. Although small bowel transplantation is the only accepted cure for CTE to date, transplantation should not be the first line of treatment given the varied natural history of the disease, with some patients showing significant improvement over time. For example, a CTE patient from Malta was reported to have a successful pregnancy [23]. Apart from the manifestation of the primary intestinal symptoms, patients with a syndromic form of CTE develop extra-intestinal symptoms [24]. A subset of CTE patients has been reported to develop ophthalmological symptoms, including superficial punctuated keratitis [16,25], cataracts [25], and corneal erosions [26]. Though rare, another subset of patients have atresias such as anal, intestinal or choanal atresia [16,26,27,28]. Moreover, CTE patients are also reported to have other signs or symptoms, including skeletal dysplasia [15], cholestatic liver disease, chronic arthritis [5,29], bone and dermatological abnormalities [16]. Arthritis is more common in patients with EPCAM mutations who have extra-intestinal manifestations, whereas ophthalmological symptoms and atresia are more common in patients with SPINT2 mutations [12]. These findings may help us in understanding the pathophysiology of this disease.

### 1.2. CTE Genetics

In 2008, utilizing genetic investigation using single nucleotide polymorphisms in a family with two children diagnosed with CTE, homozygous (or compound heterozygous) mutation in the EPCAM gene was identified as causative of CTE [10], consistent with autosomal recessive inheritance. Since then, many additional disease-associated mutations have been identified [11]. In 2018, a comprehensive study of CTE mutations was undertaken, evaluating the previously reported mutations and performing genotype-phenotype correlation [11]. Forty-two different EPCAM mutations were reported in the 2018 study, and since then, 1 novel mutation has been reported [11,30]. These mutations lead to chromosomal deletions, splicing, frameshifts, truncations and missense mutations. Patients with frameshift mutations or truncation in the EPCAM gene are reported to have a more severe disease phenotype, as seen in patients with c.499dupC EPCAM mutation [11]. Among the 42 distinct reported mutations of EpCAM that cause CTE, most result in the expression of mutant EpCAM [11,31]. In patients harboring these mutations, EpCAM is mutated or truncated, and the protein is decreased in abundance and/or mislocalized, possibly due to reduced stability [10]. SPINT2, a serine protease inhibitor, is alternatively mutated in a subset of patients with intestinal pathology consistent with CTE and extra-intestinal syndromic features [16]. Given the recent advancement in genetic technologies and improved access to whole-genome testing, patients may be identified by genetics even prior to the identification of mucosal findings.

### 1.3. CTE Models

Studies utilizing various in vivo, ex vivo and in vitro generated CTE models with EpCAM, or SPINT2 mutations reveal insights regarding the onset of the disease phenotype. Several models with SPINT2 mutations, which encodes mutant HAI-2, showed evidence of syndromic CTE and alteration of intestinal homeostasis [32,33]. Mutant HAI-2 results in the decrease/loss of EpCAM through matriptase activity [34,35], which ultimately leads to the onset of a CTE-like syndrome. Thus, the mechanistic activity for SPINT2 mutation derived CTE directly or indirectly depends on the loss of EpCAM expression. Several other studies have investigated CTE via EPCAM mutation or knockout in mice or cell lines [36,37,38]. An EPCAM knockout mouse had dysregulated E-cadherin and β-catenin in the enteric mucosa leading to crypt-villi disorganization [36]. Another study using mutant EpCAM mice, based on a CTE patient mutation, demonstrated growth retardation and CTE-like pathology, including impaired barrier function and decreases in the tight junction proteins zonula occludins-1 (ZO-1) and occludin [37]. A study with mutant EpCAM intestinal organoids revealed alterations in differentiation in addition to barrier and permeability dysfunction in tight junction proteins [39]. Moreover, data suggest that deficiency of EpCAM in a colonic cell line is accompanied by ion transport and barrier defects [37]. Given the inherent difficulty in studying pediatric patients and tissues, these models provide an important platform for prior and future investigation of this disease.

## 2. Role of Intestinal Homeostasis in CTE Pathogenesis

Disruption of intestinal epithelial homeostasis is at the forefront of CTE development. Many studies have revealed that EpCAM plays an important role in the maintenance of intestinal epithelial homeostasis in mice and humans [34,36,38,40]. Epithelial homeostasis is maintained through a dynamic equilibrium between intestinal epithelial cells (IECs) and the gut mucosal barrier (both physical and chemical). CTE disrupts various aspects of this homeostasis: cell differentiation [39,41], barrier [37], cell–cell junction [10,42] and structural composition [43]. The present review will discuss each aspect in the context of CTE to understand the pathogenesis of this disease.

### 2.1. Role of Intestinal Cell Differentiation

In the intestine, a distinct genetic differentiation program categorizes undifferentiated intestinal epithelial cells into absorptive cells and four different secretory cells: goblet cells, Paneth cells, enteroendocrine cells and tuft cells. All of these cells are differentiated from intestinal stem cells that reside in the crypt niche. On the other hand, intestinal stem cells maintain cellular homeostasis by continuously renewing to generate intermittent cells, known as transit-amplifying cells, which undergo terminal differentiation and maturation onto villi tips [44]. Studies in CTE murine intestinal tissue showed increased proliferation index when compared to its normal counterparts, and it was proposed that intestinal tissue compensates for epithelial dysfunction leading to enhanced proliferation with crowding of enterocytes [38]. Secretory cell differentiation is found to be impaired in both CTE enteroids [39] and CTE murine intestines [41], leading to decreased goblet cell and Paneth cell populations in CTE enteroids, patients, and murine intestines [39]. Data suggests that in CTE, intestinal cell differentiation is biased toward absorptive lineage differentiation through Notch signaling at the expense of Atoh1-mediated secretory lineage differentiation. Further, it was shown that Notch signal γ-secretase inhibitor treatment (DAPT) could rescue key secretory cell marker expression in a CTE enteroid model [41]. The causal mechanism behind altered cell differentiation cascade and how abnormal cell differentiation impacts CTE disease outcomes is still not known. The above studies indicate that altered cell differentiation in CTE plays an important role in its pathogenesis.

### 2.2. Role of Defective Enterocyte Function

Focal epithelial cell crowding consisting of densely packed enterocytes resembling tufts is the characteristic feature of CTE. CTE is classically considered to be caused by defective barrier function rather than defective enterocyte function [45]. However, many of the symptoms that lead to severe intestinal failure are also indicative of defective absorptive enterocyte function. Some studies suggest that the enterocyte brush border appears normal, and the expression of brush border markers remains unchanged in CTE [46]. Other studies report that the enterocyte brush border is affected in CTE, resulting in a partial disappearance or decrease of the brush border component [43]. Along with brush border, the structural components of enterocytes including ezrin, villin, apical polarity protein (crumbs 3) and the cell polarity complex (protein kinase C and par-3) are found to be mislocalized in EpCAM depleted Caco2 cells, further emphasizing alteration in epithelial cell polarity in CTE [43]. Altered localization and decreased expression of ion transporters NKCC1, but unchanged CFTR, has been reported in CTE mice and an EpCAM knockout model indicating some ion transporters are compromised in CTE [37]. This observation is further supported in the most recent report demonstrating key transporters such as NHE3 and glucose transporters, SGLT1 and GLUT2, are affected in a CTE murine model [41]. Alteration in expression and localization of ion transporters and tight junction proteins explains the secretory nature of diarrhea found in CTE, whereas changes in glucose transporters explain osmotic diarrhea seen in these patients [3,18]. The reason why the expression of some membrane transporters is altered while the others are unchanged needs to be deciphered. The explanation may lay in regulatory molecules or the trafficking of the ion transporters [47]. Moreover, apart from ion/glucose transporters, other key components of the brush border machinery, glycoside hydrolases (maltase, sucrase and lactase), that help in digestion of disaccharides are also found to be disrupted in CTE biopsies [43] and CTE mice [41], indicating involvement of malabsorption in this disease. These findings provide a rationale as to why parenteral nutrition is often the only reliable source of nutrients in these patients and the clinical observation that enteral feeding has been shown to worsen CTE [3]. The combined effect of decreased glucose transporters and disaccharidase expression result in compromised caloric uptake in this disease, which may contribute to weight loss and failure to thrive, common features of CTE.

### 2.3. Role of Defective Barrier Function and Altered Cell–Cell Junction

The most studied aspect of CTE, which provides an explanation for the severe intestinal failure and diarrhea, is the defective barrier function and disruption of cell–cell junctions. Since EpCAM is localized to tight junctions (TJ), Adherens junctions (AJ), and the lateral membrane of intestinal epithelial cell lining, mutations of EpCAM/loss of EpCAM significantly affect the cell–cell junction, which directly leads to disruption in barrier function [40]. Studies support the fact that EpCAM is essential, not only for homotypic cell–cell interaction [48], it also associates with cell adhesion molecules and regulates TJ, AJ, desmosomes and hemidesmosomes [37,38,40,49]. E-cadherin is an important cell adhesion player that regulates key TJ proteins, disruption of which compromises barrier function [50,51]. However, *how* EpCAM and E-cadherin complement each other is still not conclusive. Some data suggest EpCAM may act as an antagonist of E-cadherin [52,53], while studies in a Zebrafish model reveal EpCAM and E-cadherin tightly interact for an enhanced effect. They maintain enveloping layer integrity, which is equivalent to the epithelial layer in higher animals, suggesting they complement each other [54]. The exact role of E-cadherin in CTE disease pathogenesis is still controversial. Some reports demonstrate that the expression pattern of E-cadherin is normal in tissue from CTE patients [42] and in EpCAM mutant mice embryos [40]. Decreased EpCAM expression does not reportedly change the expression and localization of β-catenin and E-cadherin in T84 cells [55]. On the contrary, another report showed that both β-catenin and E-cadherin expression and localization is disrupted after birth in an EpCAM knockout mice model [36], suggesting it may be a secondary effect of EpCAM mutation. On the other hand, studies in EpCAM depleted amphibian embryos show decreased E-cadherin and α-& β-catenin levels at the post-transcriptional level [56]. In vitro studies also confirm altered E-cadherin expression in EpCAM depleted Caco-2 cells, suggesting both the adhesion molecules to be coordinately regulated. Unlike adherens, the role of EpCAM on TJ regulation is well studied and correlates with CTE pathogenesis. Loss of functional EpCAM, as seen in CTE, decreases the expression and alters the localization of claudin-7 [34,37,38] because the EpCAM transmembrane and intracellular region are responsible for interaction with claudin-7 [57]. In CTE, the absence of EpCAM substantially reduces the recruitment of claudin-7 to TJs, leading to loss of TJ formation [40]. Along with claudin-7 other claudins, 2, 3, 7, and 15, are also found to be decreased in the intestine of EpCAM mutant mice [40] and claudin-1 in EpCAM knockout T84 and Caco-2 cells [55]. Claudin-15, on the other hand, is responsible for paracellular permeability of Na+ and causes glucose malabsorption [58], which is another disease symptom of CTE [41]. EpCAM mutant mice also have decreased TJ proteins Zo-1 and occludin [37]. Cortical tension that helps in the localization of TJs is suspected to be disrupted in CTE due to an imbalance of EpCAM, claudin-7 and E-cadherin [49]. AJs also play an important regulatory role for the establishment of TJs [59], suggesting a combination role in CTE that leads to impaired barrier function, cell–cell junction disruption and ultimately to defective apical junctional complexes (AJC). AJCs, in turn, play a pivotal role in the maintenance of cell polarity and tissue architecture [60]. Thus, alteration in both tissue architecture and epithelial cell polarity as seen in CTE [43] strongly implicate a role of AJCs in CTE pathogenesis, possibly contributing to tuft like structure formation. The loss of EpCAM-dependent cell–cell adhesion at bicellular junctions in CTE leads to tricellular junction hypercontractility due to accumulation of myosin IIa/IIb [43]. The changes in apical-basal contractility in turn leads to abnormal increase of apical domain and finally epithelial dysplasia [43,61]. Moreover, the role of tricellular junction in pathogenesis of CTE and maintenance of IEC homeostasis is further established with a study in insect midgut that showed the importance of tricellular contacts in maintaining the intestinal barrier function, preventing terminal differentiation and increasing intestinal stem cell proliferation [62], all of which are associated with CTE. Lastly, desmosomes, another critical cell structure, are also found to be altered in CTE. The length and number of desmosomes between the epithelial cells has shown to be increased and distorted in CTE patients and CTE mice [38,42]. The intestine of the CTE mice revealed sporadic irregularity with crowding and lengthening of desmosomes [38] which supports the finding of expanded desmoglein in CTE patients [42]. Although, the role of desmosomes in CTE pathogenesis is not very clear, it can be speculated that due to imbalance between AJs and cortical tension, desmosomes are altered and mis-localized and result in loss of epithelial integrity. The increased desmosome length and number in CTE may also arise as a result of compensatory behavior where desmosomes are trying to make up for the loss of the cell–cell junction.

### 2.4. Role of Cell-Matrix Adhesion

In addition to abnormalities in cell-cell interaction, cell-matrix interactions were also were found to be impaired in CTE patients, likely contributing to disease pathogenesis. The intestinal basement membrane of CTE patient biopsies was found to have increased heparan sulfate proteoglycan, collagen type IV deposition and decreased laminin in the crypt region [3,63]. α2β1 integrin distribution along the crypt-villus axis was found to be abnormal in CTE patients, where α2 integrin was found to be expressed on the basolateral membrane lining of both the villi and crypts, unlike the normal expression on the epithelial lining of the crypt, but not in villi [3,42,63]. Several studies suggest that basement membrane molecules play an important role in the development and differentiation of the intestine and epithelial-mesenchymal cell interaction [64,65,66]. Although the molecular basis for these abnormalities has yet to be determined, cell-matrix alterations may contribute to the morphologic alterations of the CTE intestine.

## 3. Probable Signaling Mechanisms in CTE Pathogenesis

In CTE, mutations in EPCAM or SPINT2 affect the stability of EpCAM protein directly or indirectly, respectively. Though the exact mechanism for CTE pathogenesis remains elusive, our current understanding of EpCAM biology expands our knowledge of CTE pathogenesis. Many EpCAM mutations result in substitutions or deletions due to abnormal splicing or frameshift truncations. In a majority of these mutations, there is a loss of EpCAM function because mutant EpCAM is synthesized without its transmembrane and intracellular domain [11]. Thus, EpCAM is undoubtedly the key regulatory component of the disease and understanding EpCAM signaling and its involvement in different cellular, and physiological aspects will strengthen understanding of the mechanism of this disease. Along with the pathogenesis that occurs due to loss of EpCAM function, some mutations alter the structural conformation of EpCAM, directly contributing to the CTE pathogenesis [11]. Given the intricate disease pathogenesis, more than one mechanism may play a role in CTE pathogenesis.

### 3.1. Endoplasmic Reticulum Stress and Unfolded Protein Response

Mutation of EpCAM in CTE results in protein conformation changes, which alter its stability and lead to proteolytic degradation, increased internalization and accumulation in the endoplasmic reticulum (ER) [67]. The misfolded protein does not leave the ER and rather accumulates, resulting in ER stress, which ultimately leads to the unfolded protein response (UPR). CTE patients and murine models demonstrate the expression of EpCAM within the cell, rather than on the cell surface [38]. Activation of ER stress-induced UPR is observed in CTE mice. Protein kinase R-like endoplasmic reticulum kinase (PERK)-mediated pathways are severely affected with activating transcription factor 6 (ATF6), and partial inositol requiring enzyme-1 (Ire1)-mediated UPR activation [68]. UPR is currently being targeted for therapeutics in several other diseases, thus making this pathway a potentially important clinical avenue for CTE patients. Studies with an in vitro model expressing several forms of mutant EpCAM that cause CTE demonstrated that these mutant forms are not routed to the normal plasma membrane location but rather are sequestered in the ER [31]. These studies provide further indication that the retention of mutant EpCAM in the ER is likely to occur in various CTE associated mutant EpCAM forms. Some evidence suggests that ER stress and UPR may contribute to loss of intestinal epithelial stemness [69], disrupt the intestinal barrier function and increase barrier permeability [70]. Whether UPR and ER stress have any role in aberrant cell differentiation and barrier dysfunction, as seen in CTE, is still to be seen. Moreover, since the ER is the first step of the secretory pathway [71], ER stress in CTE may lead to disrupted vesicular trafficking. Although some studies are suggestive, as some membrane proteins are inappropriately expressed in CTE [41,43], further detailed studies are needed to evaluate the role of vesicular trafficking in CTE.

### 3.2. Novel Protein Kinase C (nPKC)-Mediated Signaling

Like most cell adhesion molecules, EpCAM is concentrated at intercellular boundaries and has been shown to facilitate cell–cell interactions [52]. Thus, EpCAM signaling pathways involved in maintaining cellular structural organization would be expected to play an important role in CTE pathogenesis. Direct inhibition of novel protein kinase C (nPKC) by EpCAM has shown a general cell surface protein-mediated regulation of signal transduction [56,72]. The association of the cytoplasmic domain of EpCAM to nPKC leads to subsequent inhibition of kinase, which plays an important role in the sustainability of cellular attachments [56]. The EpCAM cytoplasmic tail motif resembles the pseudosubstrate inhibitory domains of PKCs and has a high affinity with nPKCs. Thus, EpCAM inhibition in amphibian embryo and cancer cells increases nPKC activity through the Erk pathway leading to myosin activity, loss of cadherin-mediated association and breakdown of cell and tissue attachment [56]. Furthermore, EpCAM modulates myosin contractility and myosin II by inhibiting downstream effectors of the Erk pathway such as myosin light-chain kinase (MLCK) and myosin light-chain (MLC). Thus, modulation of MLCK and MLC have important implications for the reorganization of filamentous actin (F-actin). Actin filaments are dynamic structures, and a balance of actin polymerization and depolymerization must be maintained for proper cell function [73]. Although this pathway is not fully elucidated in CTE, disorganization of contractile forces at tricellular contacts, as seen in CTE [43], or actin disorganization/clumping in CTE enteroids [39] and EPCAM knockdown T84 cells [37] may be caused by loss of the inhibitory domain of EpCAM that enhances nPKC activity due to mutant EPCAM. Further assessment of this pathway in CTE is critical to a comprehensive understanding.

### 3.3. Canonical Wnt Signaling

EpCAM is a crucial signaling molecule that is involved in various developmental and physiological functions. Regulated intramembrane proteolysis (RIP) of EpCAM results in releasing the intracellular domain of EpCAM (EpICD) by two proteolytic cleavages [74]. EpICD then translocates to the nucleus to act as a transcription factor associated with components of the Wnt signaling pathway, regulating cell proliferation and tumor formation in mice [74]. This suggests potential crosstalk of EpCAM and Wnt signaling. A positive correlation is found between the expression of EpCAM and canonical Wnt signaling in colon cancer patient tissues [75], further indicating EpCAM signaling crosstalk with the Wnt pathway. Moreover, potential clues have been reported on the liaison between EpCAM expression and Wnt signaling activation in HBV-infected hepatocellular carcinoma [76]. On the other hand, in normal tissue, EpCAM mutant zebrafish show impaired liver development similar to that seen in a canonical Wnt gene mutant model [77]. EpCAM is found to counteract the endoderm specific repression of Wnt signaling in a zebrafish model through disruption of Kremen-1 and Dickkopf-2 (Dkk-2) interaction [77]. It was shown that EpCAM, by directly binding with Kremen-1, prevents Kremmen-1-Dkk-2, mediated removal of plasma membrane lipoprotein receptor-related protein (Lrp6) [77], a well-known co-receptor involved in canonical Wnt signaling. Currently, there is no data reporting how Wnt signaling is regulated in CTE, but, as EPCAM is mutated in CTE, one could speculate that canonical Wnt signaling may be implicated in the intestinal epithelial cell differentiation defects seen in this disease [39,41]. Interestingly, the expression of Atoh1/Math1, a downstream target of canonical Wnt/β-catenin signaling molecule [78], is decreased in CTE mice and enteroids [39,41]. Atoh1/Math1 is a pivotal transcription factor needed for secretory cell lineage commitment in the mouse intestine [79]. Therefore, study of this pathway may allow for understanding of the cell differentiation defects seen in CTE.

## 4. Candidate Therapeutics

Currently, the treatment strategies for CTE are supportive and primarily focused on nutrition rather than strategies to improve defects of the intestinal mucosa. No direct therapeutics are being used to treat or ameliorate CTE or its symptoms. A GLP-2 agonist, Teduglutide, is being used in other forms of the short gut syndrome [80]. In adult studies, there are emerging reports that teduglutide ameliorates several conditions, including intestinal malabsorption, short villous length as well as dependence on parenteral nutrition, which are common disease symptoms of CODEs, including CTE [81,82]. Teduglutide has shown the beneficial effect of reduced parenteral nutrition requirements in a small cohort of children with short bowel syndrome [83], suggesting investigation in patients with CTE may be promising. Although several murine model systems exist, they have not yet been utilized to test candidate drugs.

The studies of CTE utilizing different CTE models to evaluate direction mechanistic pathway, which we have discussed earlier in this review, may provide future therapeutic options. For example, upregulation of UPR [68] suggests a candidate drug may target UPR caused by mutation of EpCAM. UPR is currently being targeted for treatment in other diseases [84]. Among the different activated UPR pathways, PERK-mediated pathways are promising to treat mutant EpCAM-mediated UPR. Another study of DAPT treatment in CTE enteroids rescues the expression of secretory cell markers [41], suggesting another potential treatment worthy of further investigation. These mechanisms need more research prior to serving as a basis for studies to treat this severe and life-threatening disease. Such studies will require detailed assessment and safety evaluations before consideration for use in patients.

## 5. Future Directions

The many reported EpCAM mutations that cause CTE and the limited number of patients harboring the same mutation seriously limit a more robust understanding of disease biology. Different EpCAM mutations may have variable effects on disease outcome; thus, more genotype-phenotype correlation studies are required. Moreover, the variability of resources available to patients in different regions may contribute to variable outcomes. Thus, there is an urgent need for unified collaboration around patients with this rare disease across the globe, starting with data collection to understand the disease’s natural history. A rare disease network or CTE registry with involvement from different parts of the world would allow for sharing knowledge, which would be useful for both clinical and biological aspects. Owing to the scarcity of the patient samples, more research and funding should be directed to develop CTE models and patient-based organoid systems. The research should be bidirectional, focusing on both basic scientific and clinical outcomes and with an ultimate aim to develop and assess therapeutic strategies.

## 6. Conclusions

It has been nearly 25 years since the first recognition of congenital tufting enteropathy and over a decade since the identification of the genetic etiology of the disease. Despite this, there is little improvement in the management of this disease due to the lack of complete understanding of the pathobiology and clinical aspects. This review article focused and summarized the important, but relatively less explored, pathogenesis and disease biology of CTE and its probable connection with its genetic etiology “EpCAM”. The current review provides evidence that CTE pathogenesis is more than an alteration in cell adhesion and barrier defects. The pathogenesis involves intricate overlapping mechanisms that include defects in epithelial cells and enterocyte function ranging from enterocyte disorganization, impaired enzymes and metabolism, defective epithelial trafficking/polarity, and altered cell differentiation. Although the pathogenesis is still not fully elucidated, this knowledge provides avenues for understanding and possible therapeutic options to consider for patients with this devastating disease.
